# Effect of the Addition of Soybean Residue (Okara) on the Physicochemical, Tribological, Instrumental, and Sensory Texture Properties of Extruded Snacks

**DOI:** 10.3390/foods11192967

**Published:** 2022-09-22

**Authors:** Aunchalee Aussanasuwannakul, Chowladda Teangpook, Witcha Treesuwan, Kassamaporn Puntaburt, Pisut Butsuwan

**Affiliations:** 1 Department of Food Chemistry and Physics, Institute of Food Research and Product Development, Kasetsart University, Bangkok 10903, Thailand; 2 Department of Food Processing and Preservation, Institute of Food Research and Product Development, Kasetsart University, Bangkok 10903, Thailand; 3 Department of Nutrition and Health, Institute of Food Research and Product Development, Kasetsart University, Bangkok 10903, Thailand

**Keywords:** food waste, valorization, gluten free, plant based, legume, formulation, optimization, mixture design, extrusion, tribology

## Abstract

An extrusion process was used to improve the physical and textural characteristics of an extruded snack supplemented with soybean residue (okara). An extreme vertices mixture design with a constraint for okara flour (0–50%), mung bean flour (20–70%), and rice flour (20–80%) resulted in the production of eleven formulations. The color, radial expansion index (REI), bulk density, tribological behavior, and instrumental and sensory texture of the extruded snacks were evaluated. Increasing the quantity of okara resulted in an extrudate with a darker, redder color, decreased REI, increased bulk density, and decreased crispness. The tribological pattern of the snack was determined by its dominant composition (protein, starch, or fiber) in the flour mixture, which contributed to the stability of the lubricating film under rotational shear. A principal component analysis of sensory data captured a total of 81.9% variations in the first two dimensions. Texture appeal was inversely related to tooth packing (r = −0.646, *p* < 0.05). The optimized formulation for texture preference had an okara content of 19%, which was 104% crispier and 168% tougher than an okara content of 40%. This by-product of soybean milk processing can thus be used to develop gluten-free snacks with desirable physical characteristics and texture.

## 1. Introduction

Okara is an insoluble residue that is generated during the production of soybean milk or tofu. It proves to be a great challenge for the industry because 1 kg of soybean for tofu or soybean milk production produces 1.2 kg of wet (fresh) okara, with a moisture content that ranges from 70% to 80% [1]. It has been estimated that 14 million tons of okara have been generated worldwide till 2020 [2]. By dry weight, okara contains appreciable nutrients with a high biological value, such as proteins (11–43%), total dietary fiber (TDF, 13–63%), lipids (5–25%), and bioactive compounds (primarily isoflavones, 0.5–1 mg/g), which justify its valorization [3]. Despite its high nutritional value, okara has so far been exploited only for low-value uses, such as manure or animal feed, or it is disposed of in landfills or incinerators. Its valorization remains a challenge to the growth of the soybean-based food industry. Its high perishability and the presence of antinutritional factors that cause digestion difficulties, polyunsaturated fatty acids responsible for off odors from aldehyde compounds, and insoluble dietary fibers that lead to a gritty mouthfeel make it unsuitable for supplementation in the food industry.

Okara has been used as an unconventional gluten-free, legume-based flour in bakery products that target patients with celiac disease [4,5,6]. Dietary fiber and protein are the main molecules in okara that serve both structural and functional purposes in gluten-free food development [7]. Despite its insolubility and incomplete essential amino acid profile, the fiber- and protein-rich okara is reported to have technological and functional properties that are suitable for direct utilization in new food product development [8,9,10]. The key issue associated with the development of food products from legume-based materials is that the target physicochemical qualities can only be obtained at the expense of sensory parameters [11]. For example, okara flour, at levels as high as 50%, can be added into gluten-free snacks such as cookies to improve their protein and fiber contents. However, the hardness of the cookies increases, and the whiteness index decreases in proportion to the quantity of flour added [12].

Extrusion is a processing technology that is suitable for gluten-free products, particularly those with fiber-rich flour added. This is because it employs structural modification [13]. Extrusion can increase the quantity of soluble fiber found in the flour and thus improve its nutritional quality and processing properties [1]. During the extrusion process, shear stress caused by high screw speeds and high processing temperatures causes chemical bond breakage in complex carbohydrates, which releases sugar molecules and slightly branched hemicellulose that are solubilized [14,15]. Therefore, extrusion can also provide a solution for the undesirable properties of hardened beans and improve their nutritional value by deactivating undesirable antinutrients [16]. Due to extrusion technology, okara has been used to replace a conventional flour. For example, okara at maximum 10% blended with maize flour significantly increases the viscoelasticity of extrudate [17], okara/rice cake containing 70% okara pellets was preferred, and the sample with 90% okara pellets was liked the least [18]. Combing 33.3–40% okara with wheat flour resulted in decreased insoluble fiber (≤25.5%) and increased soluble fiber (≤150%) in extrudates [19].

Therefore, the aim of the present study is to investigate the impact of using okara in combination with other grain flours to optimize the physicochemical and textural characteristics of extruded snack formulations. The objective is to describe the effect of the proportions of okara, mung bean, rice, and corn flours (dietary fiber >15% of the dry matter) on the physical characteristics (color, expansion, bulk density, tribological behavior, and texture), sensory texture, and appeal of the extruded snacks, thus presenting a proposal for the development of gluten-free snacks.

## 2. Materials and Methods

### 2.1. Materials

Sao Hai rice (*Oryza sativa* Linn, indica type), mung bean (*Phaseolus aureus* L.), and corn grit (*Zea mays* L.) were supplied by a local market in Bangkok, Thailand. The okara used was a coprocess of the Institute of Food Research and Product Development’s pilot soymilk processing line, for which soybean (*Glycine max* L.) was supplied by Doi Kham Food Products Co., Ltd., Bangkok, Thailand. On the same day, fresh okara separated from processing was tray dried at 100 °C for 4 h until the moisture content fell below 10%. It was then pulverized using a pin mill, resulting in a particle size of between 30 and 50 mesh. The dried ingredients were thoroughly combined using a mixer (KitchenAid, 5K5SS model, Benton Harbor, MI, USA).

### 2.2. Formulation of Extruded Snack

An extreme vertices mixture design using Minitab 15 (Minitab Inc., State College, PA, USA) was used to generate formulations (Table 1). The proportions of the three components were constrained as follows: okara (0 to 50% *w/w*), mung bean (20% to 70% *w/w*), and rice (20% to 80% *w/w*). This restriction resulted in 11 design points. For each formulation, okara, mung bean, and rice were added up to 100%, and these grains constituted 79.5% of the total formulation. Calcium carbonate and corn grit were fixed at 0.5% and 20%, respectively. Direct expanded snacks are primarily made from corn grit because of its favorable yellow color, unique corn flavor, and good puffing properties [20]. Several works of research have proved that partial substitution of corn grit with other grains leads to improvement in the technological properties and economic benefits of the extruded snack [21]. Due to this, the current study set the corn grit content to 20% in the flour mixture to provide the snack’s puffing characteristic. The control used solely (100%) corn grit without any substitutions.

### 2.3. Extrusion Process and Sample Preparation

The extrusion process was carried out using an intermeshing corotating twin screw extruder (Hermann Berstorff Laboratory, ZE25 X 33D model, Germany) with a barrel length-to-diameter ratio of 870:25. This extruder comprises of 7-barrel parts terminating with a 24.5 mm-thick die plate with one circular die hole (diameter 3.0 mm). The raw material mixture was fed into the extruder using a volumetric twin screw feeder (K- Tron soder AG5702, type 20, Switzerland), and water was pumped into the ingredients for the required moisture content (15%). The temperature profiles of the 7 heating zones of the extruder barrel extending from the feeder towards the die were as follows: 37 °C, 55 °C, 121 °C, 132 °C, 164 °C, and 111 °C. The other operating conditions were set as follows: screw speed: 400 rpm, feed rate: 210.83 g/min, water rate: 19–26 g/min, feed moisture: 15–17%; and melting temperature: 152–155 °C. After extrusion, the samples were dried in an electric oven at 80 °C for 10 min and packed into aluminum foil bags at room temperature. Some extruded snacks were pulverized to 50 mesh size, and their physical properties were then examined. Intact samples were also subject to instrumental texture and sensory analyses.

### 2.4. Chemical and Physical Properties

#### 2.4.1. Proximate Composition

The proximate composition analysis of the flour followed the methods set out in [22]. The hot air oven method was used to determine its moisture content [23]. Ash content was measured by ignition at 550 °C, as was defined in AOAC 942.05 [24]. The quantity of protein was estimated using a semiautomated Kjeldhal apparatus on nitrogen bases. The crude fiber content was determined using alkali treatment. Fat content is the solvent extractable lipid content present in the sample, and this was determined using the AOAC 996.01 method described in [25]. Starch was quantified with the AOAC 996.11 method, as described in [26].

#### 2.4.2. Color

Instrumental color analysis followed the methods set out in [22]. The color of the extruded samples was analyzed using a HunterLab XE-Spector colorimeter (Hunter Associates Laboratory, Reston, VA, USA). Color was expressed as L*, a*, and b* values. The intensity of white to black colors was denoted as the L* value, redness to greenness ranged from a* to - a* value, and yellowness to blueness ranged from b*to - b* value in the Hunter Meter. The equipment was adjusted using a standard white tile (porcelain) before sample testing [27].

#### 2.4.3. Radial Expansion Index

The radial expansion index (REI) of the extrudates was calculated based on the ratio of the diameter of the die to that of the extrudates. The diameter of the extrudates was measured using a vernier caliper, and the REI was calculated using Equation (1), according to the method described in [28].

(1) $$\mathrm{REI}\;=\frac{\mathrm{Diameter}\;\mathrm{of}\;\mathrm{sample}\;\left(\mathrm{mm}\right)}{\mathrm{Diameter}\;\mathrm{of}\;\mathrm{die}\;\left(\mathrm{mm}\right)}$$

#### 2.4.4. Bulk Density

The bulk density (BD, g/L) of the extrudates was determined using the volumetric displacement method [29]. Extrudates were weighed (g) and put in a 1 L beaker, and millet seeds were then added to fill up the beaker. The extrudates were taken out and the volume of millet seeds was measured (L). The BD was calculated according to Equation (2).

(2) $$\mathrm{BD}\;=\frac{\mathrm{weight}\;\mathrm{of}\;\mathrm{sample}\;\left(g\right)}{\mathrm{volume}\;\mathrm{of}\;\mathrm{millet}\;\mathrm{seeds}\;\left(L\right)}$$

#### 2.4.5. Digital Image and Optical Microscopy

Images of the extruded snacks were captured using a digital camera (Sony, NEX-3 model, Japan) with a dimension of 4592 × 3056 and resolution of 350 × 350 in RGB color space. The microstructure of the snacks was examined using a light microscope (Olympus, BX511F model, Tokyo, Japan) at 4× magnification.

### 2.5. Tribological Analysis

**Figure 1:**
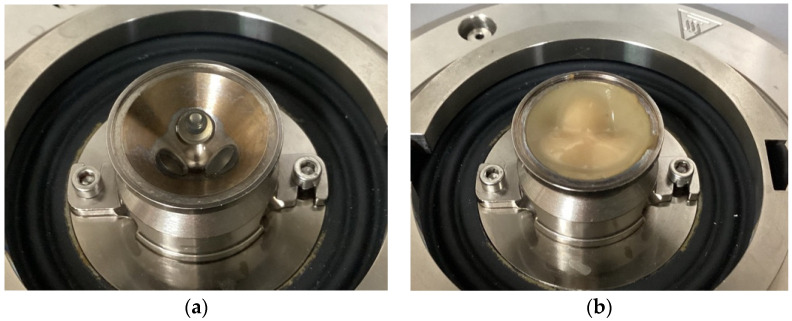
Detailed view of the sample holder with PDMS pins: (**a**) empty; (**b**) sample after measurement.

The lubrication properties of the samples were measured using a ball-on-three-pins tribo-rheometry (MCR 302 Rheometer, Anton Paar GmbH, Graz, Austria) with some modifications to [30]. For each test, around 3 g of the sample was gently loaded into the sample holder and spread out to cover the three stationary polydimethylsiloxane (PDMS) pins fixed into it (Figure 1). Measurements were performed at 37 °C with a constant normal force of 1 N. The friction coefficients between the rotating 0.5” soda-lime glass ball and the three stationary, cylindrical PDMS pins were recorded to measure their rotational speeds, ranging from 0.01 to 1000 mm/s, using RheoCompass^TM^ software (Version 1.30, Anton Paar GmbH, Graz, Austria).

### 2.6. Instrumental Texture Analysis

**Figure 2:**
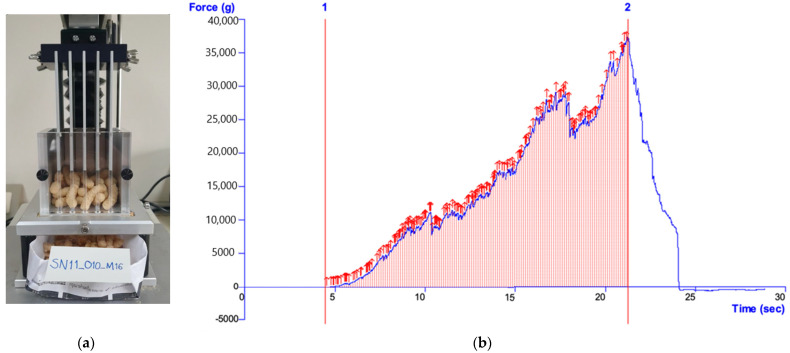
(**a**) Instrumental set up of the fracturability test consisting of a 5-blade Kramer Shear Cell mounted to a TA-XTplus^®^ Texture Analyzer; (**b**) Schematic representation of a typical force-deformation curve for crispness (peak) and toughness (g.sec).

The fracturability of the extruded snack samples was analyzed using a 5-blade Kramer shear cell mounted on a Texture Analyzer (Model TA-XTplus^®^, Texture Technologies Corp., Scarsdale, NY, USA) equipped with a 50 kg load cell (Stable Micro System’s application study, REF: SNK1/KS5). The snacks were removed from their packets just prior to testing and weighed out into equal portions (9.3 g). This amount was sufficient to fill the shear cell to 50% capacity. After loading the sample into the shear cell, the probe was set to travel at a crosshead speed of 2.00 mm/s and a distance of 45 mm. As compression proceeded, fractures could be observed as a series of force peaks. The number of major peaks over the 10 g force threshold was considered an indication of ‘crispness’. At this specified distance, the area under the curve was noted to be an indication of sample ‘toughness’. Data was recorded and analyzed using the Texture Exponent software (version 3.0.5.0; Stable Micro System Ltd., Godalming, Surrey, UK). The representative curve is shown in Figure 2.

### 2.7. Sensory Analysis

All subjects provided informed consent for inclusion before participating in the study. The study was conducted in accordance with the international guidelines for human research protection, and the methodology was approved by the Kasetsart University Research Ethics Committee (COE No. COE65/026). Sensory tests for texture preferences of the 11 extruded samples were carried out by 30 untrained volunteer panelists who were habitual consumers of extruded foods and cereal. A 7-point hedonic scale was used (1: extremely dislike to 7: extremely like). This number of untrained panelists met the criteria for the reliability of preference-testing results [23].

The sensory profiling panel comprised of 12 panelists (3 males and 9 females between 20 and 60 years old). Panelists were employees of the Institute of Food Research and Product Development, Kasetsart University, Bangkok, Thailand, and had previously been recruited for the Sensory and Consumer Research Unit’s trained panel based on their sensory acuity, discriminating ability, motivation, and availability. Panelists had been trained in sensory descriptive analysis techniques for snack products following conventional procedures [31] in addition to our in-house method, which was described in our previous study [32]. The attributes generated during the training session are shown in Table 2. Each sample treatment, coded with three random digits, consisted of five pieces of extruded snack (about 2.5 g). They were presented to the panelists in a clear, press-seal plastic bag to prevent them from absorbing moisture [33]. A balanced, incomplete block design was used for the presentation of the samples to the panelists to prevent sensory fatigue [34,35]. Natural water was provided as a taste neutralizer between products. Evaluations were made on a 7-point category scale ranging from 0 to 6.

### 2.8. Statistical Analysis

Statistical analysis was obtained via analysis of variance (ANOVA) followed by Tukey’s test. The results, expressed as mean ± standard deviation, were considered to be statistically significant if *p* ≤ 0.05. Different letters indicate significant differences in the results (*p* ≤ 0.05). The analyses were replicated at least three times. In order to obtain a better insight into the relationship among sensory attributes in snack products, principal component analysis (PCA) and Pearson product–moment correlation analyses were performed according to Aussanasuwannakul et al. [36] with some modifications using Minitab (version 15, Minitab Inc.; State College, PA, USA).

## 3. Results and Discussion

### 3.1. Chemical and Physical Properties

#### 3.1.1. Proximate Composition

Table 3 shows the approximate composition of flour used in the formulation of extruded snacks. It can be seen that flour from legumes (okara and mungbean) generally contained higher protein contents than flour from cereal grains (rice and corn grit). The later demonstrates higher starch contents. The chemical composition of okara flour used in the current study is close to that reported in existing literature [3].

#### 3.1.2. Color

**Figure 3:**
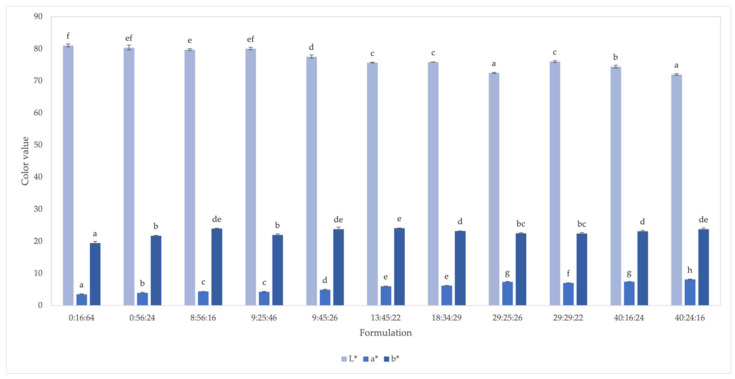
Color of extruded snacks with different proportions of flours (%okara, %mung bean, %rice). ^a–f^ Means with different letters within the same response are different (*p* < 0.05; n = 3).

Figure 3 shows the results of the evaluation of the color parameters, brightness (L*), red–green (a*), and yellow–blue (b*). It demonstrates a significant difference between the treatments. Color values were found to vary significantly between the different formulations: L* = 72–81, a* = 4–8, and b* = 20–24.

**Figure 4:**
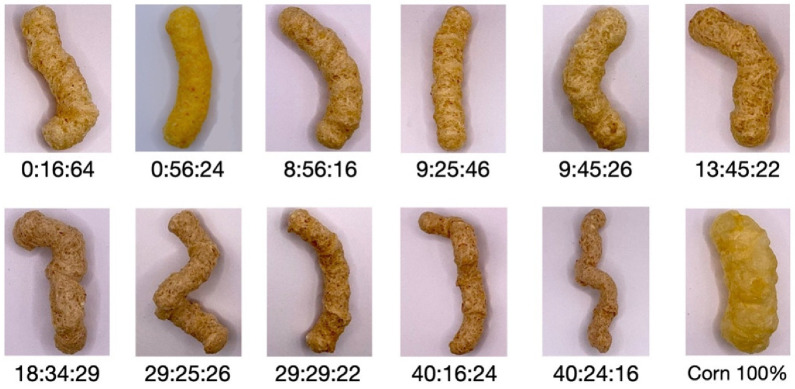
Extruded snacks with different proportions of flours (showing the ratios of okara: mung bean: rice) representing up to 80% of the formulation. One has corn as the only flour ingredient (100%).

Increasing okara powder in the formulation resulted in darker and redder snacks (Figure 4). The lowest L* value (72.51) and highest a* value (7.4) was observed in formulation 29:25:26 (Figure 3). According to [37], the color of extrudates made from agricultural by-products progressively intensifies as the quantity of by-product increases. Color changes could occur due to pigment degradation in the by-products, the Maillard reaction, and oxidation during extrusion [38]. The influence of processing temperature on the color of soy-based snacks with 13% okara was reported: The fried product was darker than the baked product (L = 50.98 vs. 53.90, *p* < 0.05; [39]).

In the formulations with relatively low levels of okara (0–9%), increasing mung bean flour from 16 to 56% significantly increased the b* (yellower) value. The color is affected by the protein content, test color, pigment, flavonoids, etc., of the grains. In mung bean flour, [40] found that L*, which represents brightness, was negatively correlated with ash content and was further affected by protein and damaged starch content. Changes in b* value, on the other hand, were related to native pigments such as uranidin, brown pigment, and flavonoids. Differences in the color index might also be caused by factors such as the degree of dispersion of the bean powder or the shape of the particles [41]. Change in the product color also resulted from the okara particle size. A smaller particle size demonstrated higher L*, lower a*, and lower b* values, which resulted in a product with a relatively lighter, greener, and bluer color [42]. As compared to the size range of 387–805 μm, okara with smaller particle sizes of 190 μm showed the highest L* value and lowest a* and b* values at 88.15, 0.39, and 12.3, respectively [42]. Okara powder used in our current study at 420 μm (40 mesh), as compared to 387 μm used by Wang and others [42] (L = 86.28, a = 0.93, b = 14.4), had a color value of L = 83.08, a = 3.31, and b = 20.22, producing an extruded snack with decreased lightness and increased reddish and yellowish coloration (L = 71.98, a = 8.12, b = 23.80; formulation 40:24:26). This was possibly due to the dilution effect and heat treatment.

#### 3.1.3. Radial Expansion Index

**Figure 5:**
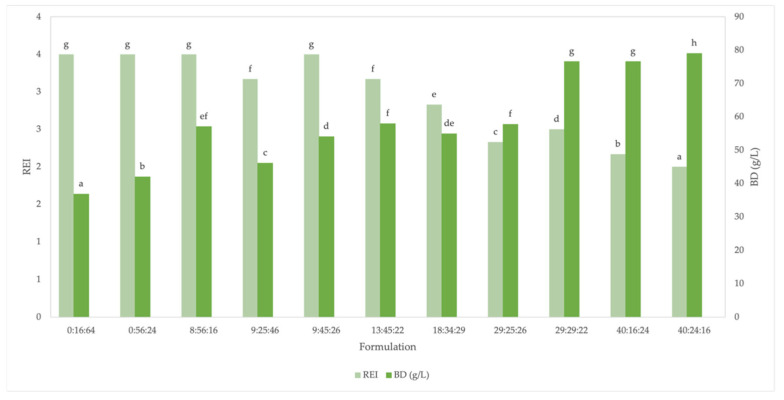
Radial expansion index (REI) and bulk density of extruded snacks with different proportions of flours (%okara: %mung bean: %rice). ^a–g^ Means with different letters within the same response are different (*p* < 0.05; n = 3).

Figure 5 shows a significant difference between the values of the radial expansion index (REI) in the extruded snack samples. Formulations with a lower okara content (0–9%) presented higher values (3.5); these formulations included 0:16:64, 0:56:24, 8:56:16, and 9:45:26. A significant decrease in REI (2–2.8) was observed when the okara in the formulation was at least 18%; these were 18:34:29, 29:25:26, 29:29:22, 40:16:24, and 40:24:16.

Expansion is the consequence of several events involving both food material and process parameters. Although starch plays a major role in expansion, other ingredients (e.g., protein and lipids) act as diluents [43]. High fiber and protein content are known to lead to decreased expansion. The lower expansion index values observed in the current study can be attributed to the fact that okara and mung bean contain both high amounts of fiber (60.75% and 15.2%, respectively) and protein (26.52% and 20.19%, respectively). The degree of fiber solubility also affects the REI. In extruded multigrain snacks, negative effects on the expansion ratio are primarily due to fiber-enriched ingredients [44]. In line with our observations, soy may not exhibit any influence on the expansion of extrudates when the proportion of soy in the blend is low (5–15%). Insoluble fiber might retain water in the matrix during extrusion cooking, thus hindering the generation of steam [45]. Furthermore, insoluble fibers tend to be stiff compared to starch-based polymers, which can inhibit expansion [46]. The dietary fiber component of the okara used in this study was primarily insoluble (96.7% according to our unpublished data [47]). Martin et al. [48] observed that although expansion was directly proportional to the starch content, a sectional expansion index decreased for all extruded snacks made from pulses and pseudocereals as the protein content increased from 30% to 70%.

**Figure 6:**
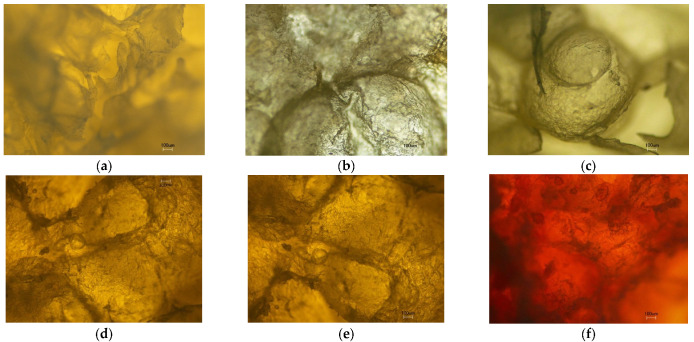
Optical microscopy of the cellular structure of extrudates at 4× magnification, arranged in the order of their starch content, from highest at 79% to lowest at 42%: (**a**) 0:16:64, (**b**) 100% corn, (**c**) 0:56:24, (**d**) 19:31:30, (**e**) 29:25:26, and (**f**) 40:24:16.

Extrusion cooking not only causes product expansion, but it also causes microstructural modification [49,50]. The microstructure of extruded snacks was found to depend on the size of the cell and its organization [38]. The degree of structure expansion positively correlated with the porosity of the cellular structure, with small, well-distributed air bubbles, compared to the less-expanded samples that presented fewer and bigger air bubbles [51,52]. Figure 4 shows the outer structure of the extruded snack captured using a digital camera. The microscopic studies (Figure 6) show the changes in the interior structure of the snacks at different flour compositions, with the expansion of the materials indicated by the presence and size of air vacuoles, void spaces, and expanded starch granules, etc. These formulations were selected from both ends of the spectrum representing the key determining factor of snack quality of interest (okara content) at two levels that could capture the underlying variation in their chemical composition; formulations with okara (29:25:26 and 40:24:16) and without (0:16:64 and 0:56:24). 

These images also reinforce the data presented in Figure 5, which show that the snacks with the lowest REI (2; formulation 40:24:16) were those with fiber = 28.70% and protein = 17.78%, whereas starch = 42% presented a more compact structure with greater interior porosity. On the other hand, a higher REI (3.5) was observed in the formulations with relatively higher starch content (0:16:64 and 0:56:24; starch = 66–79%, fiber = 3–9%, Table 4). High expansion is primarily dependent on starch content in the raw materials to be extruded. Rice flour is high in starch content whereas okara flour adds more protein to the extrudates, and therefore, the addition of okara flour causes a significant reduction in the REI. Small, expanded starch granules along with small, expanded air vacuoles and some intact starch granules were observed in the micrographs of the extruded okara-mung bean-rice-corn snack (Figure 6f). In the puffed potato-soy snack, the maximum expansion ratio (3.69), characterized as having maximum expanded porous structures with larger cracks and smaller pits, was obtained with 10.31% soy flour blended with potato flour [53].

#### 3.1.4. Bulk Density

Bulk density (BD) is an index of the extent of puffing. It is negatively correlated to the expansion ratio [9,54]. The BD of the okara in the range of 0–9% was 47 g/L and increased on average to 73 g/L in formulations with 29–40% okara, whereas REI decreased from 3 to 2, respectively (Figure 5).

Generally, high-expansion, low-density products are expected to be desirable to consumers [54]. Extrudates with a higher REI have pockets of air that give them an expanded structure. These types of extrudates have a lower bulk density because of their lower mass-to-volume ratio [45]. In the extruded corn snack, the piece density (0.38–0.41 g/cm^3^) increased whereas the REI (3.08–3.66) decreased as supplementation with soy flour increased from 20 to 40/100 g [55]. Figure 5 shows the density values apparent in the extruded snacks. The formulations 0:16:64, 0:56:24, and 9:25:46 presented the lowest density value (41.71 ± 4.68 g/L) among the grain combinations. The opposite effect occurred with increasing okara in the mixture: 29:29:22, 40:16:24, and 40:24:16 (77.46 + 1.40 g/L).

In general, we observed that the BD increased as the okara content increased. A similar result was reported by [54,56]. Starch–protein interactions are likely to have played an important role in density by disrupting the continuous starch matrix and thus reducing the extensibility of cell walls [57]. In our study, okara and mung bean added protein to the mixture, whereas rice and corn grit provided starch. The same levels of okara, mung bean, and rice contributed significantly to changes in the REI and bulk density of the snack.

### 3.2. Tribological Properties

**Figure 7:**
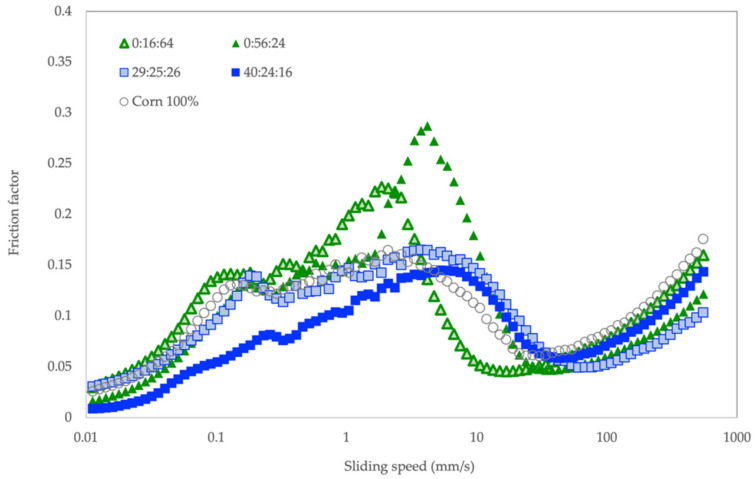
Friction factor at different sliding speeds for selected extrudate formulations with different proportions of flours (%okara: %mung bean: %rice).

Tribological properties of pastes obtained from select extrudates were determined in order to understand the effect of flour composition on friction and lubrication between interacting surfaces within the oral environment in relation to texture and mouthfeel. Three typical transitions that divide friction, or Stribeck curve into boundary regime, mixed regime, and hydrodynamic regime, can be identified from our dataset (Figure 7).

At initial sliding (below 0.1 mm/s), the friction values of all samples increased linearly with increasing speed in the absence of lubricant. Up to approximately 1 mm/s, a boundary layer began to develop. At this point, a proportion of 40:24:16 with fiber as the key component showed a relatively low friction value compared to other extrudates. According to [58], extrusion helps improve the mouthfeel of cereal and legume flours by decreasing the crude fiber content and starch retrogradation. Extrusion is an extreme mechanical treatment that that can improve the extrudate’s water binding ability by destroying the structure of dietary fiber and liberating hydrophilic hydroxyl groups of cellulose and hemicellulose [58]. Lower levels of soluble fiber friction between interacting surfaces were observed in the current study between 0.01 and 1 mm/s for the formulations with relatively higher fiber contents. The friction profile of the extrudate 40:24:26, which was made from flour mixture with 29% crude fiber (Table 4), was clearly separated from the rest (Figure 7) from the beginning until the sliding speed reached 3.7 mm/s.

A mixed regime, in which film lubrication began to develop and friction decreased with sliding speed, was observed at a broad speed range between 3 and 10 mm/s. The early onset of this mixed regime was observed in starch-based extrudates without okara (0:16:64, 0:56:24, and 100% corn). The higher peak friction that was observed in extrudates 0:16:64 and 0:56:24 (starch = 66–79%, Table 4) as compared to corn (77%) might be caused by the addition of mung bean. It is understood that mung bean contains starch with a relatively high amylose content, which constantly increases viscosity during shearing [59]. In extruded rice noodles, 5% mung bean starch could cause a decline in swelling power and a resistance against shearing while cooking [60]. Considering that the speed of the human tongue is around 20 mm/s [61], we observed a pattern similar to the mixed regime speed range. At 20 mm/s, friction values differed significantly between extrudates with and without okara, in descending order, as follows: 40:24:16, 29:25:26, 100% corn, 0:16:64, and 0:56:24 (Table 5). This difference suggests the effect of okara on the mouthfeel of these extrudates.

The final transition, the hydrodynamic regime, was observed between 50 and 100 mm/s. The hydrodynamic film was fully formed and the two contact surfaces were well separated. Friction was in the range of 0.052 to 0.13, and no differences were observed between these samples.

All extrudate samples exhibited S-type or typical Stribeck shapes that were related to the starch content [62]. According to Pang et al. [62], the flattened curves observed in extrudates with okara might have been caused by their dominant protein component due to the quantities of soybean and mung bean. The lower friction coefficient (especially in 40:24:16) could be due to its higher lipid content from the okara flour. In general, natural plant fibers obtained from agricultural residues can be incorporated into food products successfully at levels of 10% or less [63]. Based on our results, extrusion allows okara to be used at levels as high as 40% in the food matrix if mixed with other grains to impact surface properties. The in-mouth properties of extrudates with okara may be affected by the improved water binding ability caused by soluble fibers, which thus increase the viscosity and cohesiveness of the bolus. At the same time, starch gel may play a surface-lubricating role between teeth–teeth, tongue–palate, and tongue–mucosa interfaces. However, the positive effects of okara on mouthfeel in mixed-grain snacks is limited to a certain level. Xie et al. [18] reported that the mouthfeel of the puffed okara/rice cake products containing 70% okara pellets was preferred, but the one with 90% okara pellets was liked the least.

### 3.3. Textural Properties

**Figure 8:**
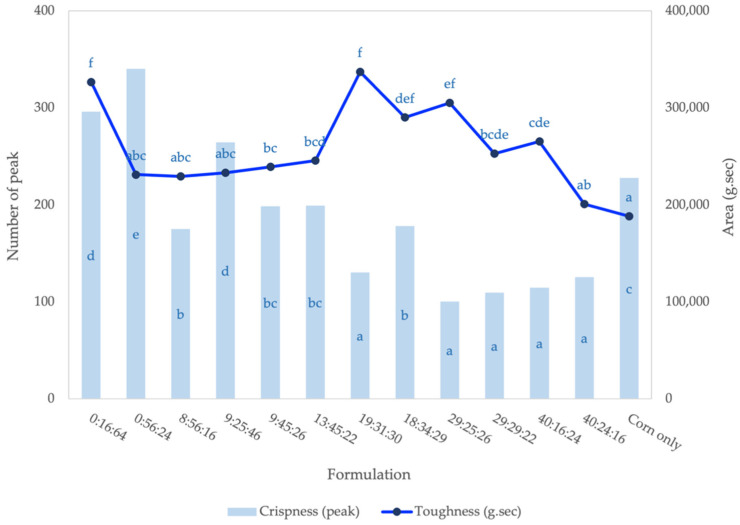
Crispness (primary axis) and toughness (secondary axis) of extruded snacks with different proportion of flours (%okara: %mung bean: %rice). ^a–f^ Means with different letters within the same response are different (*p* < 0.05; n = 3).

The current study determined the crispness of the extruded snack, with fracture peaks using bulk shearing to indicate the structure’s porosity and fracturability. Toughness (area under the curve) was determined by shearing under compression. An extruded snack is crisp if it is liable to fracture when subjected to stress. That is, it should have little tendency to deform (or strain) before fracture. We observed, in our fiber-based snack, that crispness was inversely related to toughness (Figure 8).

The crispness of an extruded product is directly related to its expansion, which, in turn, is influenced by the type of ingredients used. Lower crispness values were observed in formulations 29:25:26, 29:29:22, 40:16:24, and 40:24:16 (average=112.44 ± 10.52 peaks) with either equal proportions of grains or increasing okara content. Our observation is in line with Kanojia et al. [64], who found that the presence of okara in blend ratios (rice:okara) from 70:30 to 90:10 contributed to decreasing crispness values and increasing extrudate hardness. In sorghum-based extruded products, an increase in hardness from 96.48 to 112.77N, with levels of soy meal flour (49% protein and 24% carbohydrate) from 0 to 20%, could be attributed to a protein–starch interaction that reduced expansion [54].

We found that as the levels of okara in the mixture increased, the crispness of the extrudates decreased. A comparison of formulation 0:16:64 with 40:24:16 in Table 4 shows that increasing okara to 40% caused the crispness value to decrease by half. This decreased crispness could be a result of the increase in protein (by 2 times) and fiber (by 8 times) and the decrease in starch content (by 2 times) in the mixture. Higher protein levels in cereals decrease the expansion of the final product [65]. The incorporation of chickpeas into the rice flour provided poor texture to the extrudate. An increase in chickpea content (0–20%) led to a decrease in product expansion and an increase in bulk density, shear, and breaking strength [66]. The poor expansion and texture observed with the increased inclusion of chickpeas in the mix may be attributed to the high protein and dietary fiber content of chickpeas as compared to rice [65].

The same comparison was made between formulations without okara. The formulation 0:56:24, which had higher mung bean and lower rice quantities relative to 0:16:64, showed the most crispness due to its high protein content. The fiber and protein content of the grain affected the instrumental texture of the extrudate. Jin et al. [67] reported that increasing soy fiber by up to 40% resulted in less expanded extrudates with smaller air cell size, thicker cell walls, and, hence, increased breaking strength. In starch-based expanded snacks, the addition of 5–20% of soy protein concentrate led to lower expansion and higher mechanical strength [68]. The effect of adding soy protein concentrate to the expansion and mechanical properties of the extrudate was a reduction in cell size but an increase in the number of cells, which was attributed to the foaming action of proteins [68]. The more obvious compact structure (Figure 4) and the denser air pockets inside (Figure 6) that were observed in extrudates with 29–40% okara corresponded to their relatively lower crispness (Figure 7). According to Rodríguez-Vidal et al. [69], protein-enriched textured soy flour could be added to extruded snacks made from whole wheat at levels of up to 15% to produce a harder and more compact product. Therefore, compared to other texture-modifying processing technologies, extrusion allows a higher level of fiber-rich legume-based ingredients to be added.

### 3.4. Sensory Analysis

**Figure 9:**
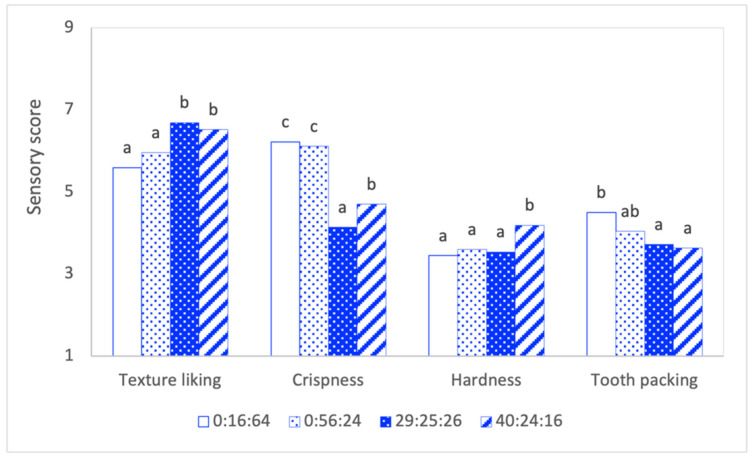
Mean score of the sensory attributes of selected snacks with different proportions of flours (%okara: %mung bean: %rice). ^a–c^ Means with different letters within the same response are different (*p* < 0.05; n = 3).

The univariate analysis of four selected formulations (Figure 9) suggested that okara can be added to fiber- and protein-rich multigrain snacks at levels as high as 40% to achieve a texture liking score of 6 out of 7. The formulations that contained 29% and 40% okara contained just half the level of starch commonly seen in regular, starch-based snacks. The preference for snacks with a high okara content is probably related to their relatively higher hardness and lower tooth packing characteristics. High hardness in snacks was attributed to the fiber content (22–29%) in the formulations, mainly from the okara. Liu et al. [70] reported that as the quantity of the okara fiber increased from 0 to 8%, the hardness of cookie dough increased significantly. Proteins and lipids in okara may contribute to lower tooth packing. According to Pang et al. [62], soybean paste with higher protein (34.98%) and lipid (16.29%) contents showed extremely low viscosity and friction compared to other grains and legumes.

Compared to starch-rich formulations, the 40:24:16 formulation is fiber-rich and thus less likely to be affected by saliva amylase that breaks down starch into smaller molecules that collect on the tooth’s surface. Furthermore, the relatively lower friction value (at 20 mm/s) observed in the tribological measurement of the snack paste could translate into a slower in-mouth bolus movement. This attracts more water and further stabilizes the lubricating film that is formed between the interacting surfaces. These factors may serve to explain the lower degree of stickiness and tooth packing of the 40:24:16 formulation observed in sensory analysis.

**Figure 10:**
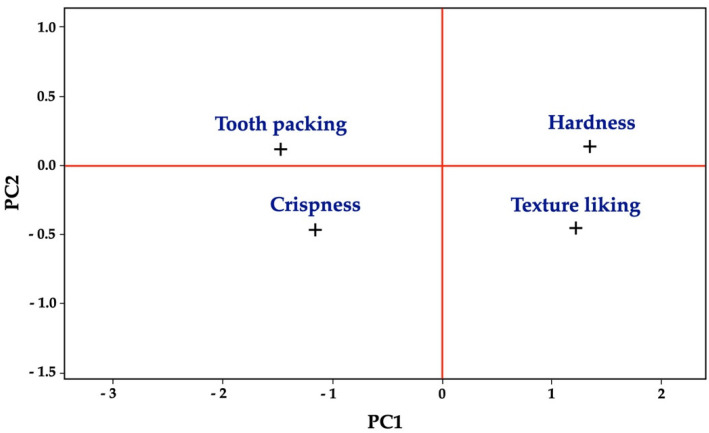
Loading plot of five sensory attributes on principal component axes. The 1st (PC1) and the 2nd (PC2) axes accounted for 65.2% and 16.7% of the total variance, respectively.

Principal component analysis (PCA) shows the relationships between the sensory variables of the different formulations of the extruded snack (Figure 10). The principal components PC 1, PC 2, PC 3, and PC 4 accounted for 65.2, 16.7, 13.9, and 4.2% of the variations, respectively. A total of 81.9% of the variations were accounted by PC 1 and PC 2. The PCA conducted, therefore, satisfies the criteria for usefulness of dimensional reduction of multivariate datasets that often retains 70–80% of the variation in the first three dimensions [71]. The loading plot shows the relationship between the sensory attributes along each axis. Texture liking and hardness were clustered in one group and were inversely related to crispness and tooth packing. This observation is in accordance with Proserpio et al. [72], who reported negative correlations between instrumental hardness, sensory crumbly (r = −0.87, *p* = 0.05), and porous (r = −0.96, *p* = 0.01) attributes. In legume-based snacks, fiber can dilute and interrupt the starch matrix and disrupt the bubble cells, leading to poorer texture (i.e., greater hardness) [73]. In high-protein extruded snacks, Kregger et al. [74] reported that the type of protein incorporated, rather than only its level, had major textural effects due to the impact of how much the snacks puffed during processing. However, these authors found that amounts of (soy) protein as high as 43% could significantly lower liking scores. A moderately negative relationship was observed for tooth packing with texture liking (r = −0.646, *p* = 0.032) and with hardness (r = −0.772, *p* = 0.005, Table 6). Tooth packing was affected by protein type [74]. These authors showed that water hydration capacities decreased with soy protein addition, which may correlate to the snack particles not dissolving during chewing.

In extruded snacks that contain various types of cereal flour (rice or wheat) and pea flour (from 60% to 90%), liking was inversely correlated with pea flour content, regardless of the cereal type [75]. These authors found that the main driver of liking was texture criteria (crispy and puffy). On the other hand, flavor perception (pea, green) constituted a barrier to acceptance. Extruded snacks with 100% pea and 15% chickpea bran were preferred to those with 100% rice by consumers (n = 72, [72]). Hedonic scores were positively influenced by crumbliness and mild flavor attributes and negatively influenced by stickiness, dryness, hardness, and, to a lesser extent, visual aspects. Since the current study focused solely on texture attributes, future studies could expand upon it to consider the perception of flavor to complete our understanding of the overall liking of these snacks.

### 3.5. Optimization of Texture Liking

The mixture design method was implemented in order to determine the optimum proportion of okara, mung bean, and rice in the dried ingredient mixture to enhance the quality of the extruded snack.

When the multiple regression analysis was analyzed, the quadratic model was found to fit well with the experimental data to represent the quality of the snack as a function of dried flour mixture (Table 7). Based on the magnitude of regression coefficients, it is suggested that okara exerted relatively greater effects on texture liking, hardness, bulk density, compression force, and a* value as compared to the other two flours. With respect to texture liking, the significance of the model was tested with the F value = 11.34 and *p* value = 0.011, suggesting that this model is a good predictor of this response. The R^2^ of 0.9165 indicated that 91.65% of the variability in texture-liking score was explained by this model.

**Figure 11:**
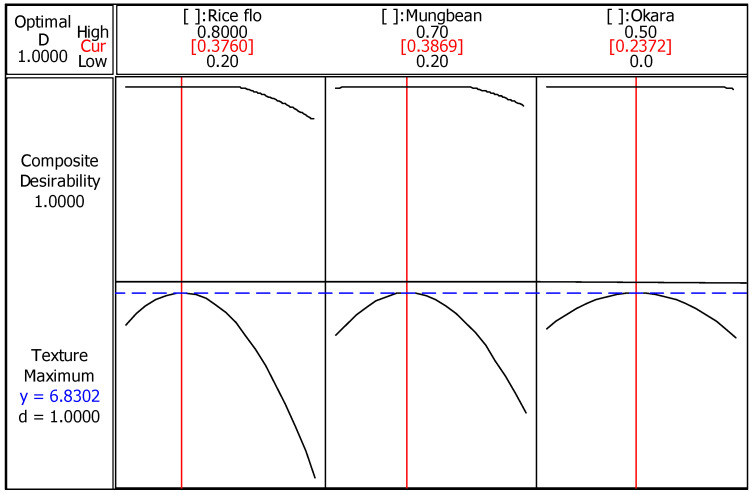
Optimal proportions of okara, mung bean, and rice flour for texture liking.

The optimization plot demonstrated the effect of dried ingredients on texture liking in the following regions of the design space: okara (0% to 50%), mung bean (20% to 70%), and rice (20% to 80%) (Figure 11). The desirability of texture liking with the optimization proportion is predicted by a high composite desirability of 1. The highest texture liking value of 6.83 was obtained using 23.72% okara, 38.69% mung bean, and 37.60% rice. Minitab software provided the optimized formulation, which contained 19% okara, 31% mung bean, 30% rice, and 20% corn. Of the eleven formulations studied, this optimized formula was relatively less crisp and more tough, with a texture value of 130.33 peaks and 3.37 x 10^5^ g.sec, respectively (Figure 8).

## 4. Conclusions

Okara, mung bean, and rice in different ratios were blended using an experimental design together with corn grit and calcium carbonate to develop a high-protein (>15%), high-fiber (22%), gluten-free snack with improved physicochemical properties, texture, and mouthfeel. The fiber–protein–starch interaction of legume and cereal-grain flours was observed, and their proportions can be optimized for color, expansion, bulk density, fracturability, surface lubrication, and sensory texture. Using an extrusion cooking process, high-protein (26.5%), high-fiber (60.8%) okara flour in the range of 29 to 40% can be mixed with mung bean flour (24–25%), rice flour (16–26%), and corn grit (20%), resulting in an extruded snack of good nutritional quality and desirable sensory perception. Okara valorization responds to the demand for plant-based food and proves to be a promising ingredient for gluten-free foods, representing a practical solution for the circular economy.

## Figures and Tables

**Table 1:** The proportion of three mixtures of grain flours generated from extreme vertices mixture design.

No.	Formulation	Okara (%)	Mung Bean (%)	Rice (%)	Corn Grit (%)
1	0:16:64	0	16	64	20
2	0:56:24	0	56	24	20
3	8:56:16	8	56	16	20
4	9:25:46	9	25	46	20
5	9:45:26	9	45	26	20
6	13:45:22	13	45	22	20
7	19:31:30	19	31	30	20
8	18:34:29	18	34	29	20
9	29:25:26	29	25	26	20
10	29:29:22	29	29	22	20
11	40:16:24	40	16	24	20

**Table 2:** List of the sensory attributes, their definitions, and the intensity scales used for profile evaluation of extruded products.

Attributes	Definitions	Scale
Crispness	Noise and force with which the sample breaks or fractures	From not crisp/soggy to very crisp
Hardness	Force required to bite through	From very soft to very hard
Tooth packing	Degree to which the sample sticks on the surface of teeth	From not sticky to very sticky

**Table 3:** Proximate composition of flour.

Flour	Protein (%)	Fat (%)	Starch (%)	Fiber (%)	Others (%)
Okara	26.52 ± 0.11 ^c^	3.32 ± 0.02 ^d^	1.12 ± 0.01 ^a^	60.75 ± 0.36 ^d^	3.18 ± 0.02 ^b^
Mung bean	20.19 ± 0.08 ^b^	1.64 ± 0.01 ^c^	52.8 ± 0.11 ^b^	15.2 ± 0.06 ^c^	3.07 ± 0.01 ^b^
Rice	6.37 ± 0.04 ^a^	0.07 ± 0.00 ^a^	86.34 ± 0.26 ^d^	0.58 ± 0.01 ^a^	0.29 ± 0.01 ^a^
Corn grit	6.52 ± 0.30 ^a^	1.2 ± 0.01 ^b^	77.37 ± 0.12 ^c^	3.28 ± 0.02 ^b^	0.3 ± 0.01 ^a^

^a–d^ Means that entries within the same column that have the same superscript or no superscript are not significantly different (*p* > 0.05; n = 3).

**Table 4:** Snack formulation, ratio of flour, and chemical composition and instrumental texture and friction values.

Formulation	Ratio of Flour (%)			Key Composition * (%)				Crispness (Peak)	Toughness (g.sec)	Friction Factor **
	Okara	Mung Bean	Rice	Protein	Fat	Starch	Fiber			
0:16:64	0	16	64	8.61	0.55	79.18	3.46	296.25 ^d^	3.27 × 10^5 b^	4.6 × 10^−2 a^
0:56:24	0	56	24	14.14	1.18	65.76	9.31	340.4 ^e^	2.31 × 10^5 a^	9.1 × 10^−2 c^
29:25:26	19	25	26	15.70	1.63	51.45	22.22	100.25 ^a^	3.05 × 10^5 b^	1.0 × 10^−1 d^
40:24:16	40	24	16	17.78	1.97	42.41	28.70	125.5 ^a^	2.65 × 10^5 a^	7.9 × 10^−2 b^

* Calculation based on the proportion of flour used; ** Determined at sliding speed of 20 mm/s; ^a–e^ Means that entries within the same column having the same superscript are not significantly different (*p* > 0.05; n = 3).

**Table 5:** Mean tribological behaviors of extrudates with different levels of flour.

Sample *	Friction Factor at Specific Sliding Speed (mm/s)						
	0.01	1	5	10	20	50	100
0:16:64	2.7 × 10^−2 c^	1.9 × 10^−1 b^	9.5 × 10^−2^	4.9 × 10^−2 a^	4.8 × 10^−2 a^	6.4 × 10^−2^	8.3 × 10^−2^
0:56:24	1.1 × 10^−2 ab^	1.3 × 10^−1 a^	1.6 × 10^−1^	8.5 × 10^−2 ab^	5.4 × 10^−2 a^	6.6 × 10^−2^	1.3 × 10^−1^
29:25:26	2.7 × 10^−2 c^	1.5 × 10^−1 ab^	1.7 × 10^−1^	1.5 × 10^−1 b^	1.0 × 10^−1 b^	5.2 × 10^−2^	5.3 × 10^−2^
40:24:16	7.8 × 10^−3 a^	1.1 × 10^−1 a^	1.5 × 10^−1^	1.3 × 10^−1 b^	8.6 × 10^−2 b^	6.0 × 10^−2^	7.2 × 10^−2^
Corn 100%	2.3 × 10^−2 bc^	1.5 × 10^−1 ab^	1.4 × 10^−1^	1.0 × 10^−1 ab^	5.9 × 10^−2 a^	6.4 × 10^−2^	8.2 × 10^−2^

* Ratio of okara, mung bean, and rice flours; ^a–c^ Means that entries within the same column that have the same or no superscript are not significantly different (*p* > 0.05; n = 3).

**Table 6:** Pearson correlation coefficient (r) between sensory attribute scores of extruded snacks.

	Texture Liking	Crispness	Hardness	Tooth Packing
Texture liking	1.000			
Crispness	−0.372	1.000		
Hardness	0.409	−0.501	1.000	
Tooth packing	−0.646 *	0.476	−0.772 *	1.000

* *p* < 0.05. n = 30.

**Table 7:** Stepwise regression equations and their coefficients of determination for responses.

Response	Regression Equation	R^2^
Texture liking	y = 3.8x_1_ + 3.53x_2_ + 4.36x_3_ + 11.72x_1_x_2_ + 6.32x_1_x_3_ + 7.99x_2_x_3_	0.9165
Crispness	y = 5.52x_1_ + 5.406x_2_ + 4.83x_3_ + 5.25x_1_x_2_ − 4.2x_1_x_3_ − 2.2x_2_x_3_	0.7183
Hardness	y = 3.09x_1_ + 3.35x_2_ + 6.3x_3_ + 1.894x_1_x_2_ − 4.72x_1_ x_3_ − 1.04x_2_x_3_	0.7405
Tooth packing	y = 4.79x_1_ + 3.55x_2_ + 4.73x_3_ − 0.01 x_1_x_2_ −4.82x_1_x_3_ −2.58x_2_x_3_	0.7168
Bulk density	y = 34.39x_1_ + 56.4x_2_ + 131.15x_3_ − 10.11x_1_x_2_ − 92.83x_1_x_3_ + 13.94x_2_x_3_	0.9212
Radial expansion index	y = 6.50x_1_ + 8.48x_2_ + 0.56x_3_ − 21.08x_1_x_2_ + 14.20x_1_x_3_ − 14.93x_2_x_3_	0.7392
Crispness	y = 4.73 × 10^5^x_1_ + 3.61 × 10^5^x_2_ – 182 × 10^5^x_3_ – 8.86 × 10^5^x_1_x_2_ +10.78 × 10^5^x_1_x_3_ + 3.27 × 10^5^	0.6132
L* value	y = 88.16x_1_ + 90.25x_2_ + 59.90x_3_ − 45.26x_1_x_2_ + 46.78x_1_x_3_ − 27.83x_2_x_3_	0.9312
a* value	y = 0.98x_1_ + 0.35x_2_ + 13.64x_3_ + 16.21x_1_x_2_ − 14.56 x_1_x_3_ + 11.23x_2_x_3_	0.9602
b* value	y = 18.54x_1_ + 24.06x_2_ + 15.02x_3_ − 0.40x_1_x_2_ + 21.35x_1_x_3_ + 19.52x_2_x_3_	0.8236

x_1_ = rice flour, x_2_ = mung bean flour, x_3_ = okara flour.

## Data Availability

The data presented in this study are available on request from the corresponding author.
